# MAGR-TCM: a knowledge graph-powered multi-agent model for intelligent home health consultation and triage in TCM gynecology

**DOI:** 10.1186/s13020-026-01485-y

**Published:** 2026-11-12

**Authors:** Ming Lv, Xu Pan, Yishan Li, Yalan Hu, Wei Xu, Li Jin, Fang Fang

**Affiliations:** 1 School of Medical Information Engineering, Anhui University of Chinese Medicine, Hefei, China; 2 School of Traditional Chinese Medicine, Anhui University of Chinese Medicine, Hefei, China; 3 School of Integrated Traditional Chinese and Western Medicine, Anhui University of Chinese Medicine, Hefei, China

**Keywords:** Traditional Chinese Medicine, Gynecological disorders, Knowledge graph, Retrieval-augmented generation, Home health consultation, Triage

## Abstract

**Background:**

Traditional Chinese Medicine (TCM) demonstrates distinct efficacy in managing gynecological disorders. However, accessing this specialized care in a home setting remains challenging due to the high cost of frequent hospital visits, privacy concerns, and a lack of immediate professional guidance. While general-purpose artificial intelligence offers a convenient alternative, the semantic gap between a patient’s colloquial symptom descriptions and formal TCM diagnostic logic—compounded by the inherent 'hallucination' risks of large language models—prevents these tools from providing safe, actionable advice. To bridge this gap, this study proposes MAGR-TCM, an intelligent home health consultation and triage model.

**Methods:**

Under expert supervision, a domain-specific knowledge graph was constructed, encompassing 10,231 entities and 32,051 relationships extracted from 741 high-quality clinical case records. Based on this foundation, we developed a Multi-Agent Retrieval-Augmented Generation (RAG) framework comprising four specialized agents designed to reconstruct the complete TCM 'Disease-Pattern-Therapeutic Principle-Formula-Medicinal' reasoning chain from informal user inquiries. This collaborative architecture simulates the expert diagnostic process to generate logical, evidence-based health guidance.

**Results:**

Experimental results demonstrate that MAGR-TCM significantly outperforms all baseline models on both RAGAS automatic evaluation and expert human evaluation. The model achieved 100% sensitivity and specificity on the safety test set, further validating its practical utility in home health settings and its ability to distinguish self-treatable conditions from those requiring urgent hospital care.

**Conclusions:**

By transitioning from black-box predictions to transparent, safe, and explainable reasoning logic, MAGR-TCM offers a technological paradigm for patient-centered home health management. It effectively mitigates generative hallucinations, making professional TCM expertise safely accessible for women’s daily self-care and preliminary triage.

## Introduction

### Advantages and challenges of TCM gynecology diagnosis and treatment

Gynecological diseases represent a significant public health concern affecting women throughout their entire life cycle, with their high prevalence and chronic recurrence contributing to an escalating disease burden. Recent data from the Global Burden of Disease Study 2021 (GBD 2021) reveals that the prevalence of gynecological diseases among women of reproductive age globally has reached 1.21 billion cases, demonstrating a trend towards younger onset [1]. This issue is particularly pronounced in China, where epidemiological investigations indicate that the prevalence rates of dysmenorrhea, Polycystic Ovary Syndrome (PCOS), and uterine fibroids have reached 45.96%, 19.04%, and 14.23%, respectively [2]. These common disorders not only impair long-term quality of life but also impose urgent demands for accessible, personalized health management that extends beyond traditional hospital settings into the daily lives of patients.

In response to this challenge, Traditional Chinese Medicine (TCM), relying on its unique theories of "Holistic Conception" and "Pattern Differentiation and Treatment," demonstrates significant value in the prevention and management of gynecological diseases. Distinct from modern medicine's focus on antagonizing lesions, TCM gynecology emphasizes personalized regulation based on constitutional differences and menstrual cycle rhythms, demonstrating definitive efficacy in alleviating chronic pelvic pain, regulating endocrine disorders, and improving assisted reproductive outcomes with minimal side effects [3]. However, TCM wisdom has long been embedded in unstructured texts such as clinical case records, ancient classics, making it difficult for home-bound patients to access and understand this tacit knowledge independently, and lineage experiences, rendering it difficult to systematically quantify and standardize [4]. For instance, descriptions in clinical case records, such as "wiry and thready pulse" or "thin yellow tongue coating," exhibit complex relationships with therapeutic interventions that uninformed users struggle to interpret. Furthermore, the TCM diagnostic process relies heavily on the personal experience and intuitive reasoning of practitioners, lacking a unified standardized pathway; consequently, existing artificial intelligence support systems often suffer from reasoning interruptions or insufficient interpretability when handling complex pattern differentiation logic [5]. Therefore, utilizing modern computational methods to structurally represent TCM knowledge and achieving interpretable, secure health reasoning for home users has become a critical bottleneck in the development of intelligent medicine.

### Technical evolution and bottleneck analysis

In recent years, breakthrough advancements in Large Language Models (LLMs) have presented new opportunities for personalized health consultation [6]. Possessing powerful natural language understanding and generation capabilities, LLMs are capable of processing unstructured inquiries from home users. However, in home-based TCM consultation scenarios where user safety and information reliability are paramount, the direct deployment of general-purpose LLMs encounters a critical bottleneck: these models are prone to "hallucinations," generating medical content lacking reliable foundations, and this uncontrollability severely impedes the safety and credibility of home health management [7].

To enhance the credibility of generated content, Retrieval-Augmented Generation (RAG) technology, which introduces external knowledge as evidentiary constraints, has emerged as a mainstream solution for mitigating hallucinations [8]. Nevertheless, in reasoning-intensive tasks dependent on complex pathogenesis and pattern differentiation logic, such as TCM gynecology, traditional Naive RAG still exhibits distinct limitations for non-professional users: first, retrieval based on vector similarity is susceptible to interference from surface keywords, making it difficult to capture deep pathogenesis logic. Second, Naive RAG, predominantly based on single-step retrieval, struggles to support the multi-step, chain-based reasoning process required for TCM pattern differentiation, often resulting in logical fragmentation that confuses home users. Third, evidence derived from document chunks in Naive RAG often appears fragmented, depriving the user of a global structured cognitive understanding of their overall health trajectory.

To further enhance structured reasoning capabilities, the GraphRAG model incorporates Knowledge Graph (KG) technology [9], utilizing graph structures to augment the model's comprehension of global information. Lightweight solutions, such as the LightRAG model [10], attempt to reduce reasoning latency while retaining structured constraints by simplifying indexing and retrieval strategies, thereby better adapting to responsive consultation scenarios. However, in case-level consultation differentiation scenarios within TCM gynecology, existing graph-augmented methods still face challenges: GraphRAG focuses on macroscopic knowledge summarization and may overlook subtle differences in TCM symptoms, thereby compromising pattern differentiation accuracy. While LightRAG improves response speed, it struggles to strictly adhere to the progressive TCM logic of "Disease–Pattern–Therapeutic Principle–Formula–Medicinal," resulting in guidance that lack a rigorous derivation process; this deficiency provides insufficient support for the safety mechanisms required for home-based users, failing to establish a stable, trustworthy closed loop. Additionally, single-agent architectures are prone to decision-making errors due to excessively long contexts or dispersed task focus when handling multi-step complex reasoning [11, 12].

The emergence of Multi-Agent Retrieval-Augmented Generation models marks a critical transition of Naive RAG systems from singular generation to collaborative, structured decision-making [12]. The advantages of this architecture are particularly prominent in high-safety-requirement fields such as home health consultation. By simulating professional consultation mechanisms, Multi-Agent RAG models decompose complex tasks into components collaboratively executed by multiple specialized agents [11]; leveraging debate and verification mechanisms among agents effectively triggers self-correction of generated content, thereby systematically reducing the risks of hallucinations and logical deviations for home users [13].

### Main contributions of this paper

Based on the aforementioned research background, this paper proposes an intelligent home health consultation and triage model for TCM gynecology, named MAGR-TCM. Supported by a TCM gynecology clinical case KG, this model constructs a Multi-Agent RAG architecture aimed at achieving accessible, traceable, and safe health guidance for home-bound users. The main contributions of this paper are summarized as follows:

1. Construction of a High-Quality TCM Gynecology Knowledge Graph for Semantic Grounding: We collected, organized, and selected 741 authentic and complete TCM gynecology clinical case records. With the participation and guidance of TCM experts, we ultimately constructed a structured KG containing the full-link logic of "Disease–Pattern–Therapeutic Principle–Formula–Medicinal," providing a solid data foundation to bridge the gap between informal patient inquiries and professional TCM knowledge.
2. Design of a Multi-Agent RAG Architecture for Secure Home Consultation: This architecture comprises four agents: the Question Analysis Agent, the Routing and Triage Agent, the Graph Reasoning Agent, and the Evaluation and Optimization Agent. Through the division of responsibilities and mutual evidentiary constraints, a step-by-step collaborative mechanism is constructed. This mechanism simulates the complete diagnosis and treatment process of TCM experts, enabling responsive correction of potential errors in each link, improving the accuracy and consistency of diagnosis and suggestion, and achieving intelligent decision-making with credible processes and reliable results, ensures that home-bound users receive health advice with credible reasoning processes and reliable safety filtering.
3. Experimental Validation of Model Effectiveness: Experimental results indicate that MAGR-TCM outperforms baseline models in both machine evaluation and human evaluation metrics, demonstrating that the model can accurately simulate expert TCM thinking, thereby providing efficient, credible, and personalized health consultation while intercepting medical risks through a safety-filtering mechanism designed for home environments.

## Methods

### Overall architecture of the MAGR-TCM model

**Fig. 1:**
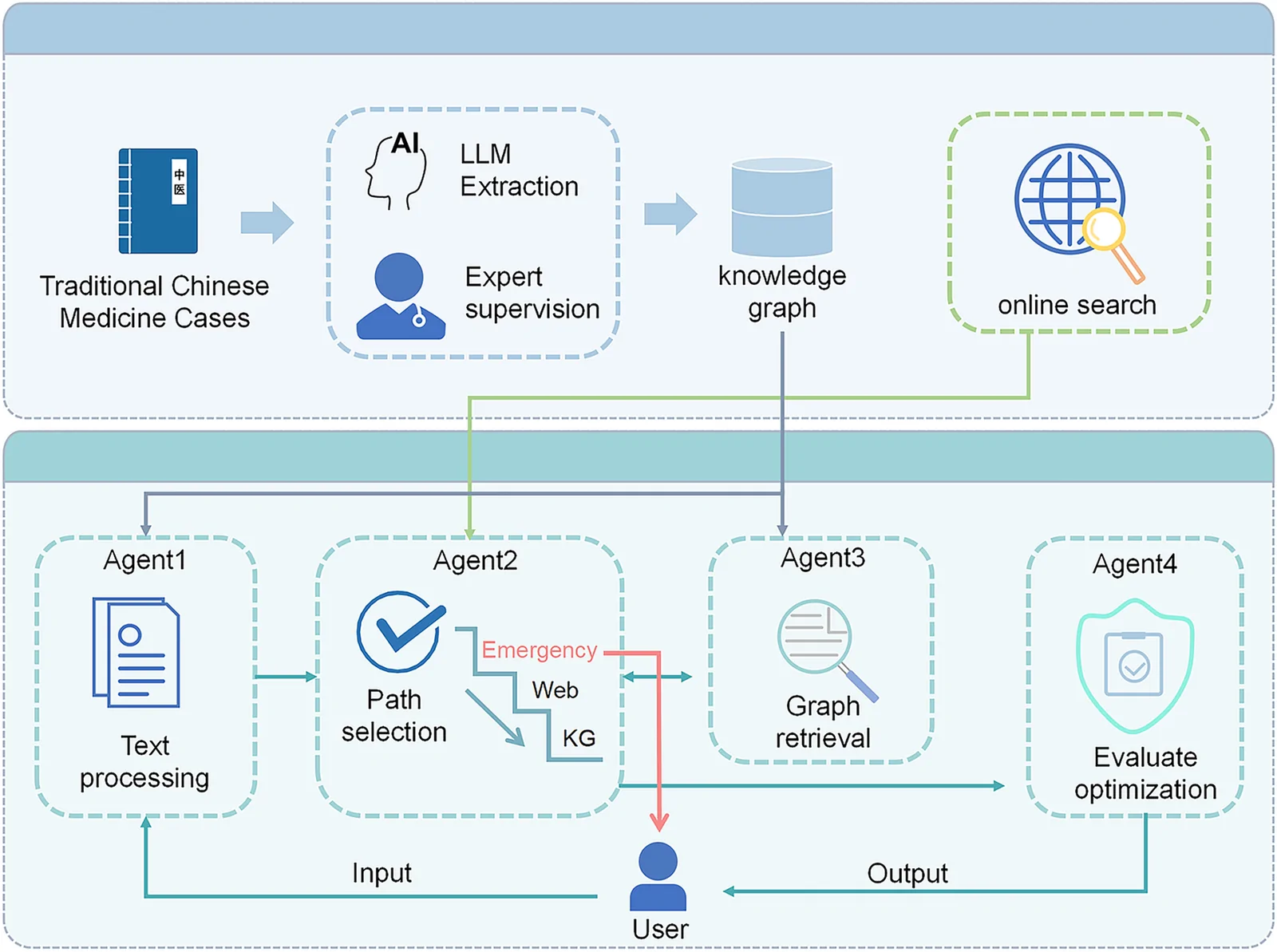
Overall architecture of the MAGR-TCM model. This figure illustrates the comprehensive framework of the MAGR-TCM model, delineating the process of constructing the TCM gynecology knowledge graph under expert supervision and the subsequent multi-agent collaborative workflow designed for intelligent clinical decision support

To address the dual challenges of structuring tacit knowledge in TCM gynecology and mitigating hallucinations in generative models, this study proposes MAGR-TCM, an intelligent health consultation and triage model that integrates a high-quality TCM gynecology clinical case Knowledge Graph (KG) with a multi-agent collaborative mechanism to support health consultation and management planning.The overall architecture of the model is illustrated in Fig. 1.

The architecture follows an "Expert-Guided Human–Machine Collaboration" workflow to extract entities and relationships from 741 TCM gynecology clinical case records, constructing a KG comprising 10,231 entities and 32,051 relationships that serves as a credible cognitive foundation for mapping user complaints to evidence-based health guidance. The multi-agent collaboration component consists of four structurally distinct agents to establish a traceable and secure reasoning chain for home settings; these agents collaborate according to a predefined workflow, imposing mutual constraints to establish a traceable reasoning chain. By leveraging hierarchical collaboration, the model progressively maps informal user inquiries to diagnosis and treatment plans that are evidence-based, logically rigorous, and auditable, thereby achieving an end-to-end trusted generation closed loop for home users.

### Construction of the TCM gynecology clinical case knowledge graph

While TCM possesses diverse data resources such as ancient literature, formula databases, and textbooks, clinical case records are characterized by the highest information density, the most complete logical chains, and the richest dimensions [14]. They serve as the most authentic bridge between TCM theory and practice and represent one of the most effective means to reveal tacit TCM diagnostic laws [15]. In the context of a home-based consultation system, these records function as a "logical gold standard" to calibrate the AI’s inferential responses to informal and colloquial user inquiries. Based on these considerations, this study established a standardized framework for KG construction, encompassing data acquisition, intelligent extraction, expert verification, and graph storage.

#### Data sources and preparation

To strictly guarantee the authority and academic reliability of the research data, this study exclusively utilized the 'Ancient and Modern Medical Case Cloud Platform' (https://www.yiankb.com/home) as the data source [16]. The data were accessed for research purposes on July10, 2025. All data utilized in this study were obtained from this publicly accessible database and were fully anonymized before access; the authors had no access to information that could identify individual participants. Therefore, ethical approval was not required for this study. This platform is a leading standardized electronic database for TCM records, independently developed and maintained by the Institute of Information on Traditional Chinese Medicine, China Academy of Chinese Medical Sciences. As a representative national-level TCM data repository, it integrates over 500,000 standardized digital clinical cases spanning from the Han Dynasty to the contemporary era, making it one of the most comprehensive and professionally curated resources in the field of TCM informatics. Leveraging this authoritative resource, we assembled a panel of three senior experts (each with > 10 years of experience) to execute a rigorous screening process. Focusing on the unstructured narratives within these clinical cases records, and given that generative models require high-density information chains to learn reasoning, we established strict inclusion criteria requiring clear diagnoses, detailed symptom descriptions, and coherent "Symptom–Pattern–Therapeutic–Principle–Formula" reasoning chains. This process prioritized the selection of the most representative and logically complete samples from the database, ensuring the construction of a high-quality Gold Standard dataset. Based on these standards, the experts retrieved and screened clinical cases for 20 representative gynecological diseases, targeting 15–40 samples per disease to ensure balance. Ultimately, 741 high-quality clinical cases were curated, providing a solid foundation for analysis. These unstructured cases serve as a "logical gold standard" for home-based consultation, providing the necessary symptomatic and therapeutic trajectories to calibrate the model's responses to informal user inquiries. The data distribution is shown in Table 1.

**Table 1:** Distribution of gynecological clinical case data

Disease Name	Count	Disease Name	Count
Threatened Abortion	40	Menopausal Syndrome	40
Dysmenorrhea	40	Adnexitis	40
Pelvic Inflammatory Disease	40	Polycystic Ovary Syndrome	40
Breast Lump	40	Infertility	40
Chronic Pelvic Inflammation	40	Endometriosis	40
Ovarian Cysts	40	Uterine Tumor	40
Menstrual Ailments	15	Preterm Labor	40
Intermenstrual Bleeding	26	Menstrual Disorders	40
Cervicitis	40	Vaginitis	40
Dysfunctional Uterine Bleeding	40	Ectopic Pregnancy	20

#### Formal definition of knowledge graph schema

To accurately characterize the diagnostic logic of TCM gynecology for home health consultation, we formally defined the KG schema. This schema consists of a core entity set $\mathrm{E}$ and a directed relationship set $\mathrm{R}$, as shown in Eq. (1).

1 $$K{G}_{Schema}=\left(E,R\right)$$

The entity set $\mathrm{E}$ comprises 11 core medical concepts extracted from clinical cases records, covering the complete diagnostic and treatment elements from manifestations to therapeutic interventions (see Table 2).

**Table 2:** Entity definitions

No	Entity label	Definition and description	Example
1	Disease	Specific disease categories diagnosed in TCM gynecology	Irregular Menstruation; Dysmenorrhea
2	Symptom	Abnormal sensations and manifestations reported by the patient or elicited during inquiry	Abdominal Pain; Lumbar Soreness; Dizziness
3	Pattern	A generalized diagnostic conclusion regarding the pathogenesis (e. g., imbalance of Yin-Yang, Qi-Blood, or Zang-Fu organs) at the current stage of the disease	Liver Qi Stagnation, Kidney Yin Deficiency
4	Medicinal	Specific herbs or medicinal materials contained within TCM formulas	Angelica sinensis [Danggui]; Paeonia lactiflora [Baishao]; Bupleurum [Chaihu]
5	Formula	Complete herbal prescriptions prescribed for specific patterns and diseases	Xiaoyao Powder; Siwu Decoction
6	Therapeutic Principle	The fundamental principles and methods guiding the treatment of the disease	Soothing the Liver and Regulating Qi; Activating Blood and Resolving Stasis
7	Tongue Manifestation	Features of the tongue body and coating observed through tongue inspection	Thin White Coating; Red Tongue with Yellow Coating
8	Pulse Condition	Pulse characteristics obtained through palpation	Thready Pulse; Wiry Pulse; Slippery Pulse
9	Physical Examination	Objective signs obtained by the physician through physical examination methods	Abdominal Tenderness; Lower Limb Edema
10	Complexion	The color and luster characteristics of the patient's face	Sallow Complexion; Pale Complexion
11	Auxiliary Examination	Laboratory or instrumental examination results from modern medicine	Ultrasound indicating Uterine Fibroids; Low Hemoglobin

The classification standards for these entities reference the Clinic Terminology of Traditional Chinese Medicine and relevant domain ontology construction specifications [17]; the relationship set $\mathrm{R}$ defines six types of directed semantic associations between entities to formally represent the reasoning path of TCM pattern differentiation (see Table 3).

**Table 3:** Relationship definitions

No	Relation label	Definition and description	Source entity	Target entity
1	Manifests	Describes specific symptoms externally manifested by a disease	Disease	Symptom
2	Indicates	Signifies that specific symptoms or signs (e. g., tongue manifestation, pulse condition) intrinsically reflect a certain pattern	Symptom, Physical Examination; Tongue Manifestation; Pulse Condition, Complexion	Pattern
3	Adopts	Denotes that a specific therapeutic principle is adopted for the treatment of a particular pattern or disease	Pattern; Disease	Therapeutic Principle
4	Selects	Indicates the selection of a specific formula when implementing a therapeutic principle or treating a particular disease	Therapeutic Principle; Disease	Formula
5	Contains	Represents the inclusion relationship, such as a disease encompassing various patterns or a formula consisting of multiple medicinals	Disease; Formula	Pattern; Medicinal
6	Suggests	Indicates that an auxiliary examination result provides diagnostic clues or suggestions for a specific disease	Auxiliary Examination	Disease

This schema is constructed top-down strictly following the TCM theoretical system, ensuring the constructed KG reliably supports the structural simulation of TCM thinking

#### Automated knowledge extraction based on expert knowledge and multi-model collaboration

**Fig. 2:**
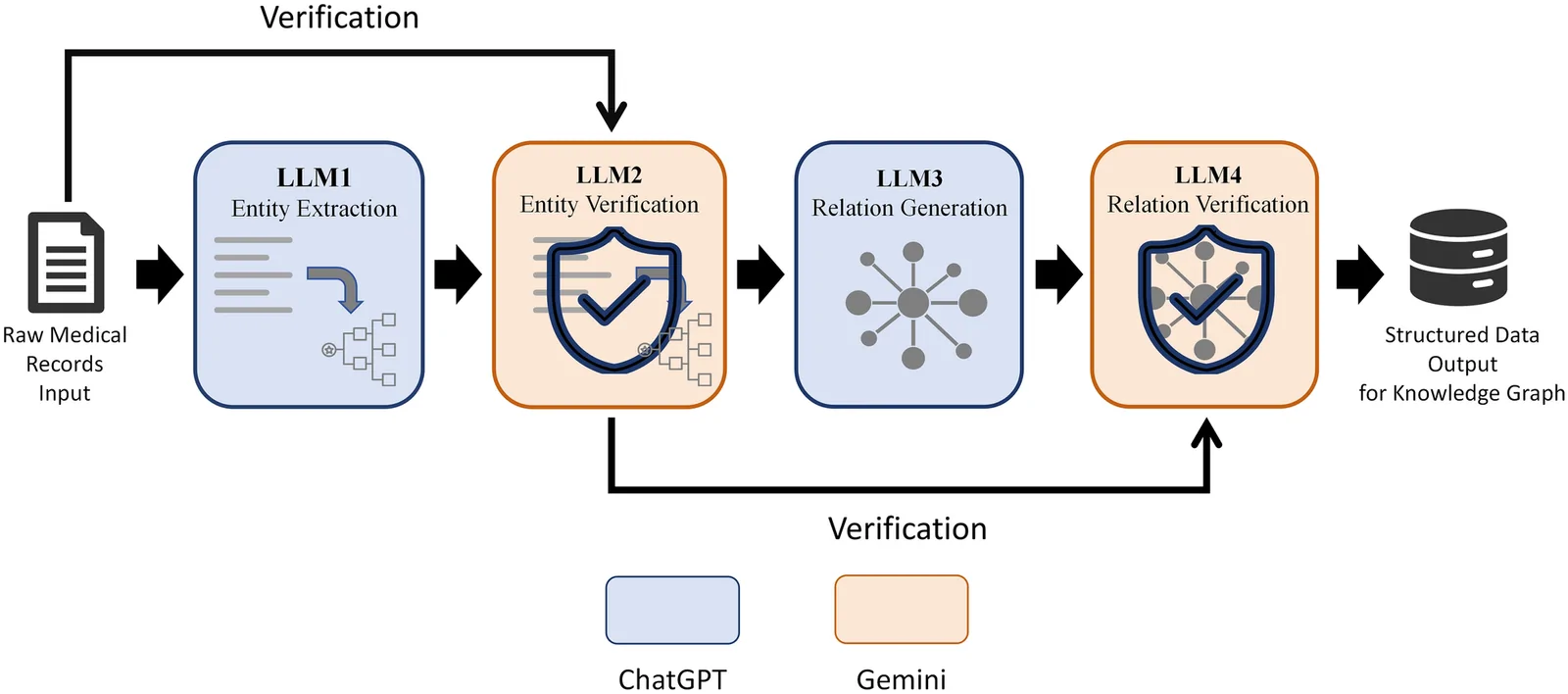
Automated knowledge extraction workflow based on the Dify platform. This figure outlines the "Human–Machine Collaborative" extraction pipeline, detailing the sequential interaction of four LLM nodes involved in entity extraction, verification, and relationship generation to ensure high-quality structured data output through a heterogeneous model strategy

To accurately extract entities and their semantic relationships from unstructured clinical case records, we designed and implemented a "Human–Machine Collaborative, Multi-Level Quality Inspection" standardization scheme. First, the expert panel randomly selected 20 cases from the dataset and performed fine-grained full-text annotation of entities using the Doccano annotation tool [18]. Subsequently, these annotated results were exported as high-quality few-shot examples. Utilizing prompt-based In-Context Learning [19], guiding an LLM to infer extraction criteria and automatically generate standardized prompts. These prompts underwent multiple rounds of expert review and refinement before being established as the unified specification for automated processing. To apply this specification efficiently across all 741 cases, we implemented an automated workflow on the Dify platform [20], comprising four interconnected LLM nodes. This architecture ensures extraction consistency through rigorous inter-node collaboration. Furthermore, to mitigate systematic bias from single models, we adopted a heterogeneous model strategy [21]. Specifically, GPT-4 was employed for entity extraction and relationship generation, while Gemini was utilized for verifying the logical consistency of entities and relationships; this dual-model cross-validation approach enhances the credibility and quality control of the results. The specific technical implementation path is illustrated in Fig. 2.

#### Expert verification and correction

To guarantee data accuracy and reliability, a rigorous expert verification and correction phase was implemented [22], aimed at systematically improving the quality of automated extraction results and ensuring compliance with TCM theory and practice standards. In this phase, all 741 structured clinical case records generated by the automated process were individually reviewed by the expert panel. Any automated deviations discovered during review, such as inconsistent relationships or missing entities, were recorded and manually corrected. The corrected data were re-aligned and underwent a final expert review to form clean data compliant with TCM pattern differentiation logic.

This human-in-the-loop iteration effectively eliminated potential systematic errors and "hallucination" risks in the automated process, significantly enhancing data credibility and value, thus laying a solid foundation for the subsequent construction and application of the high-quality KG.

#### Graph storage and visualization

**Fig. 3:**
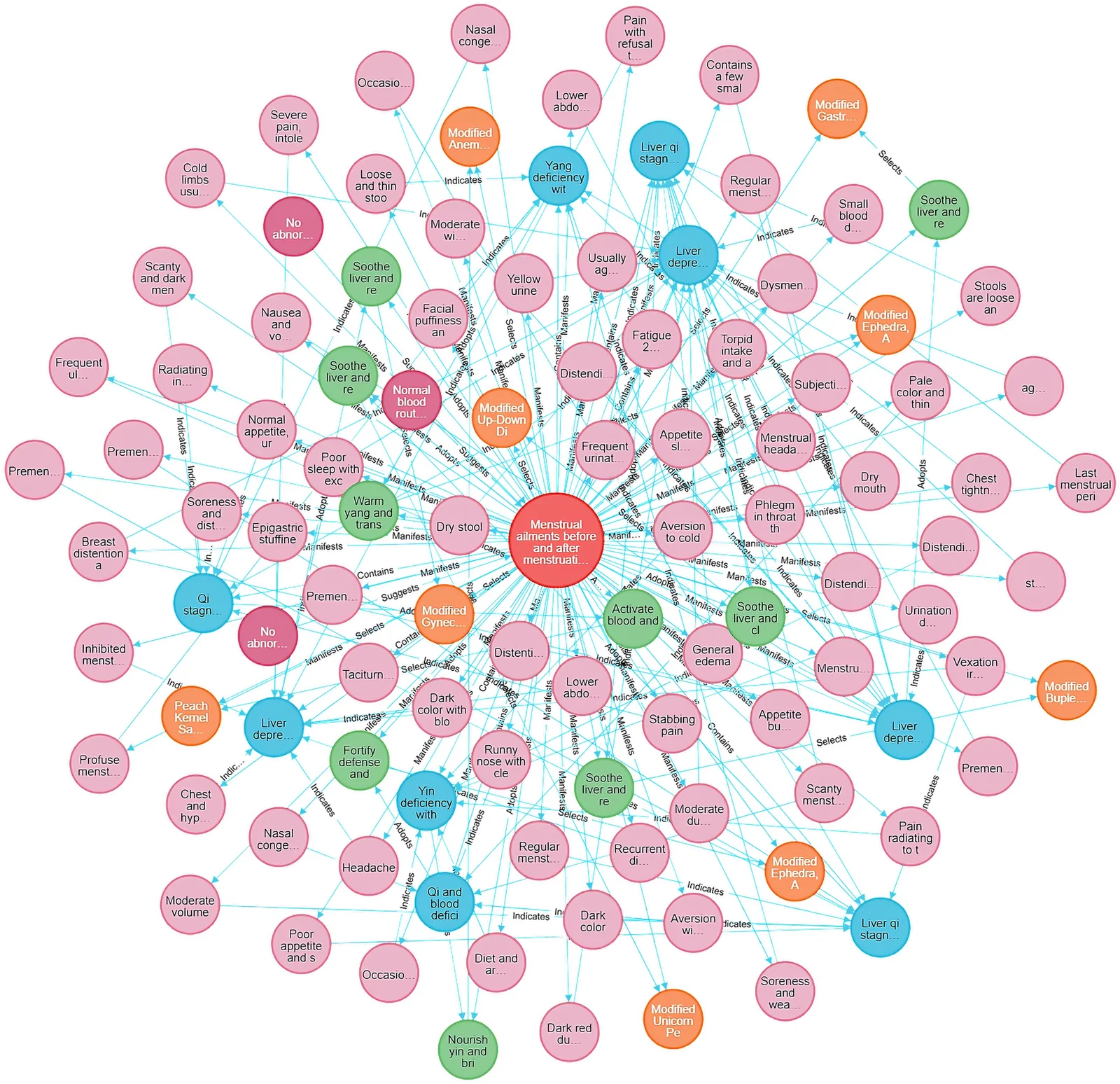
Partial view of the TCM Gynecology clinical Case Knowledge Graph. This figure presents a visualization of a segment of the constructed knowledge graph, displaying the complex semantic network and topological relationships among core medical entities in the graph database

Upon completion of information extraction and verification, the standardized data were stored as a queryable and computable KG. We utilized the Neo4j graph database [23] and developed automated scripts based on the Cypher query language to batch import the verified 10,231 entities and 32,051 relationships, thereby finalizing the construction and storage of the TCM gynecology clinical case KG. A partial view of the TCM Gynecology clinical Case Knowledge Graph is presented in Fig. 3, intuitively displaying the semantic network structure of TCM gynecological concepts.

### Multi-agent retrieval-augmented generation framework

**Fig. 4:**
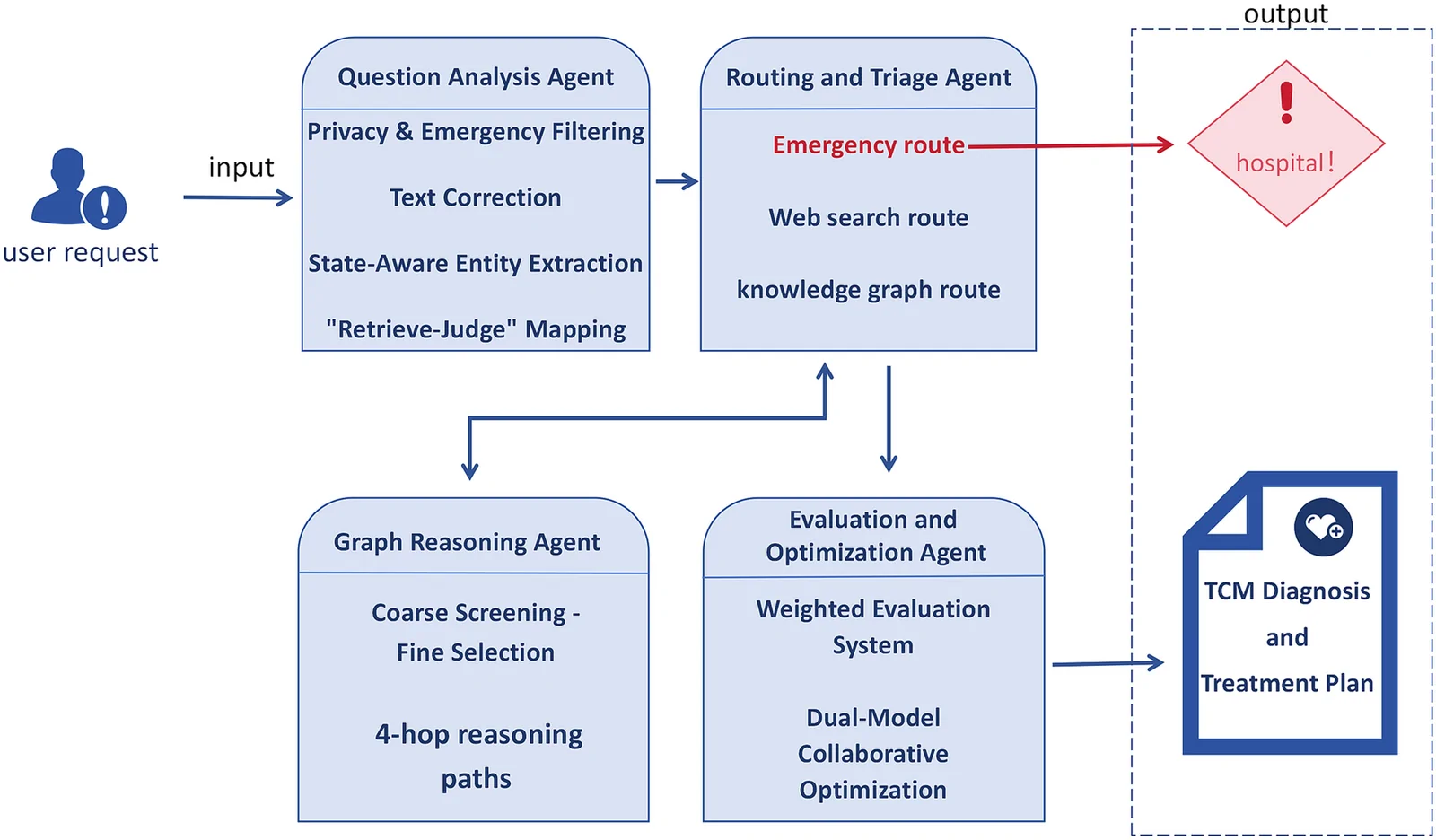
Architecture of the multi-agent collaborative mechanism for TCM gynecology diagnosis and treatment. This figure details the specific workflow and responsibilities of the four agents (Question Analysis, Routing, Graph Reasoning, and Evaluation Optimization), highlighting the dynamic routing protocols and the closed-loop safety mechanism used to generate credible, evidence-based treatment plans

Based on the constructed KG, we designed a Multi-Agent RAG framework. This framework decomposes complex TCM diagnostic decision tasks into sequential execution by four agents with distinct responsibilities. These agents are: the Question Analysis Agent, Routing and Triage Agent, Graph Reasoning Agent, and Evaluation and Optimization Agent. This multi-agent architecture simulates the TCM collaborative diagnostic mechanism, significantly enhancing accuracy, process interpretability, and logical consistency through refined task division and decision coordination. The specific workflow is illustrated in Fig. 4.

#### Question analysis agent (Agent 1)

In home settings, user input is often fragmented and colloquial; the quality of this input directly impacts the performance of downstream reasoning models. To address this challenge, we designed the Question Analysis Agent (Agent 1). This agent adopts a "Safety-First, Layer-by-Layer Cleaning" pipeline architecture, comprising a Privacy Filtering and Emergency Grading Layer, a Text Correction Layer, an Entity Extraction Layer, and a Semantic Mapping Layer based on the "Retrieve-Judge" paradigm.

(1) Privacy Filtering and Emergency Grading Layer.

Adhering to medical ethics and data security regulations, Agent 1 first executes privacy filtering on user inputs [24]. Agent 1 de-identification filter based on regular expressions is deployed. This filter performs high-precision pattern matching and masking on sensitive personal information (e. g., mobile numbers, identification numbers, and names), ensuring that such data is anonymized before entering the cloud-based LLM reasoning process.

Furthermore, guided by gynecological emergency clinical guidelines, Agent 1 incorporates a built-in "Red Flag" vocabulary containing high-risk indications such as "shock," "massive hemorrhage," and "severe pain." Upon securing data privacy, the agent immediately scans the input text; if any critical indicator is detected, the subsequent reasoning process is instantaneously terminated, and a high-priority emergency alert is transmitted to Agent 2 to safeguard patient safety.

(2) Text Correction Layer.

Following safety filtering, the input proceeds to the correction layer. Initially, the DeepSeek LLM is utilized to perform semantic analysis on the input text [25], identifying potentially erroneous terms, denoted as ${w}_{raw}$, based on context, and generating candidate corrections, denoted as ${w}_{sugg}$, thereby forming a set of potential errors and their corresponding candidates. Subsequently, a dual verification method incorporating phonetic similarity ${S}_{pho}$ and morphological similarity ${S}_{morph}$ is applied [26]. This method calculates the respective similarity values between the potential error and the candidate correction; a replacement is executed only when both values exceed their predefined thresholds, thereby preventing erroneous corrections by the LLM. The calculations for phonetic similarity ${S}_{pho}$ and morphological similarity ${S}_{morph}$ are presented in Eqs. 2 and 3, respectively.

2 $${S}_{pho}=\frac{{F}_{audio}\left({w}_{raw}\right)\cdot {F}_{audio}\left({w}_{sugg}\right)}{\Vert {F}_{audio}\left({w}_{raw}\right)\Vert \times \Vert {F}_{audio}\left({w}_{sugg}\right)\Vert }$$

where ${F}_{audio}\left({w}_{raw}\right)$ and ${F}_{audio}\left({w}_{sugg}\right)$ represent the phonetic feature vectors of the original term and the candidate correction, respectively.

3 $${S}_{morph}=1-\frac{D\left({w}_{raw},{w}_{sugg}\right)}{max\left(len\left({w}_{raw}\right),len\left({w}_{sugg}\right)\right)}$$

Here, $\mathrm{D}\left({w}_{\mathrm{r}\mathrm{a}\mathrm{w}},{w}_{\mathrm{s}\mathrm{u}\mathrm{g}\mathrm{g}}\right)$ denotes the edit distance between the original term and the candidate correction. and $\mathrm{l}\mathrm{e}\mathrm{n}\left({w}_{\mathrm{s}\mathrm{u}\mathrm{g}\mathrm{g}}\right)$ represent the lengths of the original term and the candidate correction, respectively. In this study, the thresholds were established as ${\tau}_{poh}=0.85$ and ${\tau}_{morph}=0.60$. A correction is applied only when both criteria are simultaneously satisfied, thereby preventing excessive intervention in the original text.

(3) State-Aware Fine-Grained Entity Extraction Layer.

Following text normalization in the preceding layers, Agent 1 executes the joint extraction of TCM entities and their associated states. Distinct from traditional Named Entity Recognition (NER), this layer simultaneously determines the state during entity extraction to construct a reliable evidence chain. The extraction target is formally formulated as a triplet $\mathrm{E}=\left({\mathrm{e}}_{\mathrm{i}},{\mathrm{c}}_{\mathrm{i}},{\mathrm{s}}_{\mathrm{i}}\right)$, where ${\mathrm{e}}_{\mathrm{i}}$ denotes the entity name, ${\mathrm{c}}_{\mathrm{i}}$ represents the 11 predefined entity categories, and ${\mathrm{s}}_{\mathrm{i}}$ indicates the state label. The state categories are defined as follows:

① Positive: Symptoms explicitly confirmed by the patient, serving as core anchors for downstream graph reasoning.

② Negative: Symptoms explicitly denied by the patient (e. g., "no abdominal pain"). While extracted, these entities are filtered during reasoning to prevent noise induction and logical deviation [27].

③ Uncertain: Symptoms described with ambiguity or speculation. The model automatically assigns an "Uncertain" tag, and the subsequent reasoning algorithm reduces their evidentiary weight accordingly.

To achieve these objectives, Agent 1 is initialized with a structured System Prompt containing definitions for the 11 entity types and state determination rules. This prompts the LLM to perform deep semantic analysis and Dependency Parsing, enabling the precise detection of negation markers for Negative state labeling and the resolution of ambiguous expressions for Uncertain state labeling, thereby achieving high-precision joint extraction.

(4) Semantic Mapping Based on the "Retrieve-Judge" Paradigm.

To bridge the semantic gap between colloquial user expressions and standard KG terminology, and to address the challenge of aligning high-similarity entities, we designed a two-stage semantic mapping mechanism comprising "Vector Retrieval Preliminary Screening" and "LLM Final Adjudication. "

Initially, utilizing a pre-trained Chinese semantic vector model, extracted Positive and Uncertain entities are mapped alongside standard KG terms into a unified semantic vector space to calculate semantic distance via cosine similarity. Standardized mapping is triggered for Positive entities with similarity > 0.7, while Uncertain entities are retained only at a similarity of 1.0 to ensure reliable anchors. Subsequently, the DeepSeek model is introduced as a "Semantic Judge. " It executes a rule-based binary verification function ${f}_{verify}\left({e}_{raw},{e}_{kg}\right)$ on preliminary results, strictly adhering to three medical constraints [28]:

① Anatomical Consistency: The anatomical site referred to by the entity must not shift across systems or organs.

② Temporal Unit Consistency: Period description units (e. g., "day," "month," "year") must remain unaltered.

③ Core Semantic Polarity Conservation: The pathological nature (e. g., "pain" vs. "painless") must not be reversed.

The entity mapping is adopted only when the verification function returns True; otherwise, a safety fallback mechanism is triggered to retain the original expression. This paradigm balances alignment efficiency with medical rigor, providing high-confidence entity anchors for subsequent graph reasoning.

#### Routing and triage agent (Agent 2)

We designed the Routing and Triage Agent (Agent 2), which is centered on executing a deterministic routing protocol. This protocol dynamically routes user queries to the most compliant and reliable knowledge domain, facilitating a transition from black-box prediction to process-explainable decision-making. Functioning as the model's "Central Triage Desk," Agent 2 routes requests to three mutually exclusive processing paths based on the query content: Emergency Safety Block, Web Search, or Knowledge Graph Retrieval.

(1) Emergency Safety Block.

This path represents the highest-priority logic within Agent 2.Upon receiving a "Red Flag" critical indication identified by Agent 1, Agent 2 immediately triggers the emergency blocking mechanism, terminating all subsequent retrieval and reasoning processes to output mandatory offline emergency guidance. Strictly adhering to emergency triage standards, this mechanism constitutes the ethical safety foundation of the model [29].

(2) Web Search.

This path is responsible for identifying and processing queries that fall outside the scope of traditional TCM pattern differentiation and treatment. Based on the NCCN Clinical Practice Guidelines and authoritative medical terminologies, we constructed an Out-of-Domain Terminology Repository covering terms. If Agent 2 detects any term from this repository in the output from Agent 1, it determines that the query requires reference to the latest modern medical guidelines, and the system forcibly routes the request to the LLM-based web search. Furthermore, if the valid entity set parsed by Agent 1 is empty (indicating a lack of semantic anchors), the system degrades the query to the web search module to prevent invalid graph traversal and conserve computational resources, thereby acquiring broader supplementary information [30].

(3) Knowledge Graph Retrieval and Closed-Loop Quality Control.

The system defaults to the Knowledge Graph Query Mode only when the emergency block is not triggered and valid semantic anchors are present; it then transitions to the Graph Reasoning Agent to execute deep reasoning based on the logic chain of "Disease–Pattern–Therapeutic Principle–Formula–Medicinal. "The high-order topological structure of the KG plays a pivotal role here; it not only links explicit entities but also mines latent implicit pathogenesis and medicinal associations through multi-hop paths, returning the retrieved content to Agent 2.

However, due to the sparsity of real-world clinical case data, graph reasoning may still encounter risks of path interruption or insufficient reasoning evidence. To address this, we introduced a Three-Element Integrity Detection Mechanism within the graph reasoner to parse retrieved results, ensuring the inclusion of three core elements: Disease, Pattern, and Formula. If any element is missing, the system determines the current graph reasoning as invalid and automatically triggers a fallback to the web search module for information supplementation. This closed-loop mechanism effectively prevents "null responses" or "logical fragmentation" caused by single-source limitations, significantly enhancing the model's robustness and continuity in complex real-world scenarios [31].

Ultimately, regardless of the routing path taken, Agent 2 synthesizes the content for output. If the retrieved content is a structured reasoning path from the KG or unstructured text from the web, Agent 2 performs semantic recombination to generate a response in a standard format comprising "Core Diagnosis, Differentiation Basis, and Suggested Formula," which is then passed to the subsequent Agent 4 for evaluation and optimization. Conversely, in emergency situations, the process terminates immediately following the issuance of the emergency medical alert.

#### Graph reasoning agent (Agent 3)

The Graph Reasoning Agent is the core reasoning engine of the entire framework. When the system selects the graph reasoning path, it transitions from Agent 2 to Agent 3, whose core task is to explore logical paths within the structured knowledge graph. Traditional beam search faces two limitations in complex TCM scenarios characterized by concurrent symptoms and intertwined pathogenesis: first, fixed bandwidth is easily monopolized by high-frequency paths, causing critical low-frequency clues to be prematurely pruned; second, paths spread exponentially during multi-hop expansion, introducing massive noise and reducing the signal-to-noise ratio. This study designed a "Coarse Screening—Fine Selection" dual-layer cascade architecture to form a progressive information funnel.

(1) Coarse-Grained Broad Recall.

This study designed a dynamic elastic dual-track beam search algorithm, achieving an adaptive balance between reasoning breadth and depth through elastic resource allocation and dual-track competition mechanisms. Given that Agent 1 has completed entity alignment, this step directly anchors the user's intent entities to their corresponding nodes in the KG. In subsequent retrieval, these nodes serve as starting points for expansion to achieve content mining; this precise entity alignment ensures that subsequent reasoning is strictly based on verified facts, preventing semantic drift [32]. In graph reasoning, the number of paths determines the capacity for tracking evidentiary clues, whereas traditional fixed path bandwidth settings often face severe supply–demand contradictions. This model introduces a linear scaling mechanism to dynamically calculate the total search path count, ensuring that search breadth scales adaptively with case complexity. The calculation formula is shown in Eq. 4.

4 $${W}_{stotal}=Clamp\left({W}_{base}+\alpha \cdot \left|{N}_{start}\right|,{W}_{min},{W}_{max}\right)$$

Here, $\left|{N}_{start}\right|$ denotes the number of starting anchors, Clamp represents the interval truncation function, ${W}_{base}$ is the base bandwidth, α=5 is the complexity adaptation coefficient, and ${W}_{max}$/${W}_{min}$ represent the upper and lower bandwidth thresholds. Using a baseline of 30 paths, this mechanism elastically expands by 5 paths for each additional entity, capped at ${W}_{max}=120$, thereby ensuring coverage while minimizing invalid computation [33].

To prevent resource monopolization by high-frequency symptoms and ensure minimal representation for every anchor, the model allocates a minimum guaranteed quota ${Q}_{min}$ to each starting anchor ${n}_{i}$ (Eq. 5).

5 $$Q_{\min } = \max \left( {2,\min \left( {5,\left \lfloor \frac{{C_{ref} }}{{\left| {N_{start} } \right|}}\right \rfloor}\right)} \right)$$

where $\left|{\mathrm{N}}_{\mathrm{s}\mathrm{t}\mathrm{a}\mathrm{r}\mathrm{t}}\right|$ represents the count of valid entities in the query, and *C*_*ref*_ is the total allocated path quota, set at *C*_*ref*_ = 100.This value is slightly lower than the maximum path limit ${\mathrm{W}}_{\mathrm{m}\mathrm{a}\mathrm{x}}=120$; the difference constitutes a resource buffer zone, reserving elastic space for subsequent processing steps.

Following guaranteed allocation, the remaining bandwidth is distributed through global competition based on path confidence, ensuring that core complaints receive prioritized resources for deep reasoning.

(2) Fine-Grained Semantic Selection

In complex cases, the number of paths often reaches hundreds following multi-hop reasoning. To mitigate context overload caused by path explosion, this phase extracts the most concise and relevant evidence subset.

To further identify core evidence most relevant to the user's complaint, the model integrates BGE-Reranker [34] as a re-ranking module. This module performs deep semantic interaction calculations on each "User Query – Reasoning Path" pair to derive a relevance score. The number of paths finally input into the LLM is dynamically adjusted based on query complexity (Eq. 6).

6 $$K_{final} = \min (\max (20,2 \times N_{entities} ),60)$$

where $N_{entities}$ denotes the number of entities and $K_{final}$ represents the final path count. This mechanism ensures that only the highest-scoring paths are input into the LLM.

Through this "Coarse Recall → Fine Selection" cascade strategy, Agent 3 optimizes the context signal-to-noise ratio while maintaining reasoning breadth, ensuring that the retrieved content is both comprehensive and precise.

Upon completion of graph traversal, Agent 3 outputs a set of reasoning paths generated via a 4-hop deep search. The 4-hop setting aims to precisely replicate the TCM pattern differentiation and treatment chain of "Symptom–Pattern–Therapeutic Principle–Formula–Medicinal" at the computational level. Specifically:

Start (Hop 0): Disease Symptoms (Representation Anchors).

Hop 1: Symptom → Pattern (Qualitative Determination of Pathogenesis).

Hop 2: Pattern → Therapeutic Principle (Establishment of Treatment Principles).

Hop 3: Therapeutic Principle → Formula (Specific Formula Carrier).

Hop 4: Formula → Medicinal (Final Therapeutic Intervention).

This depth represents the minimal necessary steps to cover the full trajectory from "Disease Representation" to "Therapeutic Intervention," ensuring structured and interpretable reasoning while preventing information diffusion and noise accumulation caused by excessive hops.

Following this reasoning process, Agent 3 transmits the final selected set of reasoning paths back to Agent 2.This step marks the completion of the tacit knowledge mining task; Agent 3's comprehensive retrieval mechanism provides a solid evidence-based foundation for Agent 2's generation of diagnostic responses.

#### Evaluation and optimization agent (Agent 4)

The Evaluation and Optimization Agent receives and processes the aggregated diagnostic response generated by Agent 2.Functioning as the final decision-making component of the framework, Agent 4 is designed to address the zero-tolerance requirement for "hallucinations" in home-based health consultation, where unverified medical information could lead to significant safety risks for non-professional users, this module abandons the traditional single-pass generation mode in favor of a trusted generation closed loop, comprising a weighted evaluation system and a dual-model collaborative optimization mechanism.

(1)Weighted Evaluation System.

To quantitatively evaluate the generated responses, we adopted the RAGAS evaluation framework [35], selecting four core metrics: Faithfulness, Answer Relevance, Context Recall, and Context Precision. Furthermore, we restructured the weight distribution of these metrics $\mathrm{W}=\left\{{\mathrm{w}}_{\mathrm{f}},{\mathrm{w}}_{\mathrm{r}},{\mathrm{w}}_{\mathrm{c}},{\mathrm{w}}_{\mathrm{p}}\right\}$ based on the specific characteristics of TCM diagnosis and treatment, thereby defining the Comprehensive Confidence Score ${\mathrm{S}}_{\mathrm{t}\mathrm{o}\mathrm{t}\mathrm{a}\mathrm{l}}$ as shown in Eq. 7.

7 $${S}_{total}={W}_{f}\cdot {S}_{faithfulness}+{W}_{r}\cdot {S}_{answer\_relevance}+{W}_{c}\cdot {S}_{context\_recall}+{W}_{p}\cdot {S}_{context\_precision}$$

The weights for the core metrics were assigned as follows: $w_{f} = 0.3$(Faithfulness), $w_{r} = 0.5$ (Answer Relevance), $w_{c} = 0.1$ (Context Recall), and $w_{p} = 0.1$ (Context Precision). This weighting strategy is derived from the characteristic of the TCM "Holistic Conception" [36]: in real-world scenarios, users require not only formula-based guidance derived from the KG but also holistic lifestyle advice (e.g., dietary restrictions, emotional regulation) that leverages the LLM's internalized general medical knowledge. Excessive emphasis on faithfulness metrics risks misclassifying such "benign generated content"—which is highly valuable to patients—as "extrinsic retrieval noise". The model establishes a passing threshold of ${\mathrm{S}}_{\mathrm{t}\mathrm{o}\mathrm{t}\mathrm{a}\mathrm{l}}$>0.7. Any generated result falling below this threshold is classified as a "Potential Risk Response," triggering the subsequent optimization process.

(2) Dual-Model Collaborative Optimization Mechanism.

For responses failing to meet the threshold, we introduced a Heterogeneous Dual-Model Collaborative Optimization mechanism, leveraging DeepSeek (specializing in Chinese medical reasoning [25]) and GPT-4 (specializing in logical instruction adherence [37]). The optimization process is capped at three rounds and employs an alternating dominance strategy: if $Round_{t}$ is generated by DeepSeek, $Round_{t + 1}$ is reviewed, corrected, and reinforced by GPT-4.This approach effectively integrates the strengths of both models, mitigating the cognitive blind spots inherent in a single model [38]. Should the score remain substandard after three iterations, a Selective Fallback Mechanism is triggered: both models are invoked in parallel to independently generate final responses. The system then recalculates scores, selects the response with the higher comprehensive confidence, and explicitly flags it as an LLM-generated response.

## Results

To evaluate the effectiveness and applicability of the MAGR-TCM model in complex TCM gynecology diagnostic scenarios, we designed a series of rigorous comparative experimental study. The study aims to address the following four core research questions:

1. Diagnostic Accuracy: Can the model accurately differentiate patterns and prescribe consistent formulas aligned with expert clinical logic?
2. Safety and Triage Robustness: Can the model effectively intercept medical risks and correctly triage emergency scenarios in unmonitored home environments?
3. Operational Feasibility: Does the system’s computational latency meet the practical requirements for real-world home health consultation deployment?
4. Architectural Justification: Within the multi-agent framework, Agent 3 traverses the Knowledge Graph using 4-hop depth. Is this 4-hop setting the optimal depth?

### Experimental setup

#### Test dataset collection and standardization

To objectively assess the model's generalization capability in real-world environments, we constructed an external test dataset completely independent of the training data, sourced from the publicly accessible Ancient and Modern Medical Case Cloud Platform. All case records in this dataset—covering both Cohort A and Cohort B described below—were independently collected and selected by the TCM expert panel, with no overlap with the 741 cases used for training. As all clinical case were fully anonymized prior to access, ethical approval was not required. Cohort A includes 30 novel gynecological clinical case records covering a typical spectrum of common and frequent gynecological diseases. Cohort B consists of another 30 cases specifically designed as a safety stress-test set, with 10 emergency "Red Flag" scenarios and 20 routine cases, used exclusively for evaluating the emergency detection mechanism. Each case underwent multiple rounds of review by senior experts to guarantee the completeness of disease information and clarity of diagnosis, covering a typical spectrum of common and frequent gynecological diseases. The expert panel established standard diagnosis and treatment plans for this test set to serve as the Gold Standard, enabling rigorous evaluation of the model's reasoning accuracy and consistency with logic when encountering unseen cases.

#### Baseline models

To comprehensively evaluate the performance of the proposed MAGR-TCM, we selected three Retrieval-Augmented Generation (RAG) models representing distinct technical paradigms as baselines to benchmark our model's advantages in complex TCM reasoning tasks.

Naive RAG:A classic architecture based on vector similarity [8]. Utilizing the bge-large-zh embedding model [34] for document chunk retrieval and matching, this model lacks the capability to model structured knowledge and deep semantic associations. It serves as a baseline to highlight the fundamental limitations of unstructured retrieval in addressing the TCM logical chain of "Disease–Pattern–Therapeutic Principle–Formula–Medicinal. "

GraphRAG:A graph retrieval paradigm based on community summaries [9]. While focusing on the macro-level understanding of global information, this model may suffer from insufficient granularity in micro-path reasoning for specific cases; it is included to contrast differences in graph utilization strategies.

LightRAG:A lightweight graph retrieval framework emphasizing entity co-occurrence and flattened retrieval [10]. This model was selected to verify the necessity of the "Deep Multi-Hop Reasoning" mechanism adopted in this study for ensuring the logical integrity of TCM pattern differentiation and treatment.

To ensure fair comparison, all models—including MAGR-TCM and the three baselines—utilized GPT-4 as the uniform generative foundation. During inference, the decoding temperature for all text generation nodes was strictly locked at 0.0 with top_p = 1.0.

#### Evaluation metrics

We employed a comprehensive evaluation system combining Automated Quantitative Evaluation and Expert Human Evaluation to assess model performance holistically.

(1) Automated Quantitative Evaluation.

We utilized the RAGAS framework [35] for standardized measurement of generated results, focusing on three core metrics, each scored on a scale of 0 to 1.

Faithfulness: Measures whether the generated answer strictly adheres to the retrieved context, serving as a core indicator to evaluate the model's ability to suppress hallucinations.

Answer Similarity: Utilizes an embedding-based approach to quantify the semantic and stylistic proximity between the generated response and the established standard answer.

Answer Correctness: Assesses the factual accuracy of the core clinical assertions by identifying overlapping semantic propositions via LLM-based parsing, while mathematically incorporating the embedding-based semantic similarity as a weighted component to provide a holistic correctness evaluation.

(2) Expert Human Evaluation.

To address the limitations of automated metrics in assessing medical logic, the TCM gynecology expert panel evaluated the generated treatment plans under double-blind conditions, with each metric scored on a scale of 0 to1.The evaluation dimensions included Pattern Identification Accuracy, Logical Consistency (across principles, methods, formulas, and medicinals), and Clinical Utility.

(3) Statistical Analysis Framework To assess whether observed performance differences between MAGR-TCM and the baselines are statistically meaningful, all metric scores underwent paired significance testing with family-wise error rate controlled via Holm-Bonferroni correction: paired t-tests for the RAGAS metrics and paired Wilcoxon signed-rank tests for the expert human ratings. Both paradigms adopted the same effect size metric r, to ensure methodological consistency and facilitate cross-paradigm comparison.

#### Supplementary experimental protocols

To complement the standard generation quality metrics, we designed three additional experiments: a safety stress-test on the emergency detection mechanism, an end-to-end latency measurement for deployment feasibility, and a hop-depth ablation study to empirically justify the 4-hop design choice.

1. **Safety stress-test design**
  To specifically validate the safety-filtering mechanism, we conducted a dedicated stress-test on Cohort B. This experiment evaluated the emergency blocking performance using three primary metrics: sensitivity, specificity, and false negative rate (FNR). Given that the baseline models lack dedicated emergency triage modules, their performance on this stress-test serves as a reference to highlight the necessity of a dedicated safety mechanism in home-use medical consultation model. All evaluations were conducted under double-blind conditions by the TCM expert panel, with ground-truth emergency labels pre-established for each case in Cohort B.
2. **Latency measurement**
  To evaluate practical deployability in real-world home consultation scenarios, we measured the end-to-end response latency (mean ± SD) for all models on the combined 60 clinical case records from Cohort A and Cohort B under identical hardware and network conditions. For MAGR-TCM, we further decomposed the total latency by individual agent to identify computational bottlenecks and assess whether the multi-agent architecture meets acceptable response time requirements for asynchronous home health consultation.
3. **Hop-depth ablation setup**
  To determine the minimal reasoning depth required for complete TCM clinical reasoning chain coverage, we designed a hop-depth ablation experiment on the 30-case Cohort A test set. We systematically evaluated 2 hop, 3 hop, 4 hop, and 5 hop reasoning paths, with hop count defined as the number of relation edges traversed from the starting symptom entity to the terminal medicinal entity in the knowledge graph. For each depth, we measured: (1) entity type coverage, i.e., the proportion of the five entity types along the complete reasoning chain (Symptom, Pattern, Therapeutic Principle, Formula, and Medicinal) that are covered from the input Symptom anchors—since Symptom itself is the starting point and always counted, coverage effectively reflects the reachability of the remaining four downstream types; (2) cumulative path count, i.e., the total number of distinct reasoning paths retrieved at that depth; and (3) marginal cost per 1% coverage gain, calculated as the additional paths required to achieve each incremental coverage improvement. This ablation setup enables identification of the depth at which further hops yield diminishing returns, thereby empirically justifying the 4-hop design choice.

### Results

#### Automated quantitative evaluation results

Table 4. presents the comparative results of the MAGR-TCM model against baseline models regarding RAGAS generation quality metrics.

1. Faithfulness:MAGR-TCM achieved the highest score of 0.821, significantly outperforming the baselines. GraphRAG followed with 0.766, while LightRAG and Naive RAG recorded lower scores of 0.651 and 0.590, respectively.
2. Answer Similarity:MAGR-TCM demonstrated the highest similarity (0.970). Naive RAG also exhibited a relatively high similarity score (0.952), whereas GraphRAG scored the lowest (0.809).
3. Answer Correctness:MAGR-TCM showed a significant advantage with a score of 0.909. In contrast, GraphRAG and LightRAG scored significantly lower at 0.495 and 0.510, respectively.

**Table 4:** Comparison of automated quantitative evaluation results across models

	Faithfulness	answer_similarity	answer_correctness	Average score
MAGR-TCM	0.821	0.970	0.909	0.900
Naive RAG	0.590	0.952	0.888	0.810
LightRAG	0.651	0.845	0.510	0.669
GraphRAG	0.766	0.809	0.495	0.690

#### Expert human evaluation results

To validate the model's practical utility, Table 5 presents the scoring results from the expert panel.

1. Pattern Differentiation Accuracy: MAGR-TCM achieved the highest score of 0.898 followed by LightRAG (0.734) and GraphRAG (0.521), with Naive RAG scoring the lowest (0.294).The performance gap between MAGR-TCM and the strongest baseline (LightRAG) is 0.164, while Naive RAG trails by a substantially larger margin of 0.604.
2. Consistency of Principles, Methods, Formulas, and Medicinals: MAGR-TCM scored highest (0.884).LightRAG ranked second (0.700), while GraphRAG (0.287) and Naive RAG (0.142) both fell below 0.3, reflecting fundamental difficulties in maintaining coherent “Symptom → Pattern → Principle → Formula → Medicinal” reasoning chains without structured KG guidance.
3. Clinical Utility: MAGR-TCM was rated highest (0.889), demonstrating strong practical deployability for home health management. LightRAG followed at 0.732, while GraphRAG (0.413) and Naive RAG (0.254) were rated substantially lower, indicating that their outputs lacked the structured clarity and safety assurances required for lay user comprehension.

**Table 5:** Results of TCM expert human evaluation

	Pattern differentiation accuracy	Consistency of principles, methods, formulas, and medicinals	Clinical utility	Average score
MAGR-TCM	0.898	0.884	0.889	0.890
GraphRAG	0.521	0.287	0.413	0.407
LightRAG	0.734	0.700	0.732	0.722
Naive RAG	0.294	0.142	0.254	0.230

#### Statistical validation

To assess the statistical reliability of the performance differences between MAGR-TCM and the baseline models, we conducted Bootstrap resampling (1,000 iterations) to compute 95% confidence intervals for each metric, and performed paired significance tests with Holm-Bonferroni correction to control the family-wise error rate. A unified, distribution-agnostic effect size metric—correlation coefficient r—was adopted across both evaluation paradigms. Concretely, r is derived from the standardized mean difference across the 30 paired cases, quantifying the magnitude of MAGR-TCM's advantage as a proportion of total variance.

(1) RAGAS automatic evaluation

Table 6 presents the Bootstrap 95% confidence intervals for the RAGAS metrics. MAGR-TCM's confidence intervals are narrow across all three dimensions (e.g., Faithfulness: [0.814, 0.829]; Answer Correctness: [0.902, 0.916]), indicating stable and reproducible automated evaluation scores. The CI lower bound of MAGR-TCM's Faithfulness (0.814) exceeds the CI upper bounds of Naive RAG (0.598), GraphRAG (0.773), and LightRAG (0.659), confirming non-overlapping confidence intervals.

**Table 6:** RAGAS evaluation: Bootstrap 95% confidence intervals (n = 30, 1000 iterations)

Model	Faithfulness	Answer similarity	Answer correctness
MAGR-TCM	0.821 [0.814, 0.829]	0.970 [0.964, 0.976]	0.909 [0.902, 0.916]
Naive RAG	0.590 [0.582, 0.598]	0.952 [0.944, 0.960]	0.888 [0.880, 0.896]
GraphRAG	0.766 [0.759, 0.773]	0.809 [0.802, 0.817]	0.495 [0.488, 0.502]
LightRAG	0.651 [0.644, 0.659]	0.845 [0.837, 0.853]	0.510 [0.502, 0.517]

Table 7 reports the paired t-test results. All nine comparisons between MAGR-TCM and the baselines are statistically significant after Holm-Bonferroni correction (p < 0.05). Effect sizes range from r = 0.559 (MAGR-TCM vs. Naive RAG) to r = 0.998 (MAGR-TCM vs. GraphRAG). The two comparisons yielding the smallest effects — Answer Similarity vs. Naive RAG (r = 0.594) and Answer Correctness vs. Naive RAG (r = 0.559) — correspond to the only cases where MAGR-TCM had reversals. This reflects the fact that Naive RAG can occasionally produce superficially appropriate language on simple cases, but its unstructured retrieval fundamentally limits factual reliability. GraphRAG and LightRAG, despite employing graph-based retrieval, are outperformed by MAGR-TCM by wide margins, particularly on Answer Correctness.

**Table 7:** RAGAS evaluation: paired t-tests and effect sizes (n = 30)

Metric	vs	t(29)	p	r
Faithfulness	Naive RAG	40.73	3.80 × 10^-27^	0.991
	GraphRAG	9.38	2.73 × 10^-10^	0.867
	LightRAG	31.9	3.90 × 10^-24^	0.986
Answer similarity	Naive RAG	3.97	4.31 × 10^-4^	0.594
	GraphRAG	39.35	1.01 × 10^-26^	0.991
	LightRAG	23.65	1.66 × 10^-20^	0.975
Answer correctness	Naive RAG	3.63	1.07 × 10^-3^	0.559
	GraphRAG	76.81	4.61 × 10^-35^	0.998
	LightRAG	66.79	2.61 × 10^-33^	0.997

(2) Expert human evaluation

Table 8 presents the Bootstrap 95% confidence intervals and per-case variance (σ^2^) for the expert evaluation. MAGR-TCM achieves the highest mean scores across all three dimensions (0.884–0.898) with the lowest variance (σ^2^: 0.0011–0.0013), indicating not only superior average performance but also consistent quality across heterogeneous clinical cases. LightRAG ranks second with moderate scores (0.700–0.734) and relatively low variance (σ^2^: 0.0023–0.0035), suggesting that its lightweight graph retrieval reliably produces acceptable outputs but lacks the depth of MAGR-TCM's multi-hop KG reasoning. In contrast, Naive RAG exhibits the highest variance (σ^2^: 0.0253–0.0803), with individual case scores spanning from 0.00 to 0.83, reflecting its fundamental dependence on whether the retrieval module happens to find relevant content.

**Table 8:** Expert evaluation: Bootstrap 95% confidence intervals and per-case variance (n = 30, 3-rater mean)

Metric	Model	Mean	σ^2^	95% CI
Pattern differentiation accuracy	MAGR-TCM	0.898	0.0013	[0.885, 0.910]
	LightRAG	0.734	0.0023	[0.718, 0.751]
	GraphRAG	0.521	0.0233	[0.468, 0.573]
	Naive RAG	0.294	0.0803	[0.197, 0.394]
Consistency	MAGR-TCM	0.884	0.0011	[0.871, 0.894]
	LightRAG	0.700	0.0028	[0.682, 0.718]
	GraphRAG	0.287	0.0328	[0.221, 0.351]
	Naive RAG	0.142	0.0253	[0.089, 0.199]
Clinical utility	MAGR-TCM	0.889	0.0011	[0.877, 0.901]
	LightRAG	0.732	0.0035	[0.711, 0.753]
	GraphRAG	0.413	0.0167	[0.368, 0.460]
	Naive RAG	0.254	0.0272	[0.194, 0.314]

Table 9 reports the paired Wilcoxon signed-rank test results. All nine comparisons are significant at p < 0.001 after Holm-Bonferroni correction. The Wilcoxon W values are reported as T^+^ (sum of positive ranks), with a theoretical maximum of n(n+1)/2 = 465 for n = 30. Seven of nine comparisons achieved T^+^ = 465, indicating that MAGR-TCM outperformed the baseline in all 30 cases. The remaining two comparisons (vs. LightRAG on Pattern Differentiation Accuracy and Clinical Utility) show T^+^ = 435 with 29 wins and 1 tie (diff = 0, excluded per standard Wilcoxon practice, yielding n' = 29).

Effect sizes range from r = 0.889 to 0.971, all firmly in the large-effect range. The smallest margins are observed against LightRAG (r = 0.922–0.947), reflecting the fact that both models employ graph-based retrieval and the performance gap narrows as the baseline's architectural sophistication increases. Conversely, the largest effect is against Naive RAG on Consistency (r = 0.971), consistent with Naive RAG's inability to maintain coherent diagnostic reasoning chains.

**Table 9:** Expert evaluation: Paired Wilcoxon signed-rank tests and effect sizes (n = 30)

Metric	vs	W (T^+^)	W/L	Exact p	r
Pattern differentiation accuracy	Naive RAG	465	30/0	1.86 × 10^-9^	0.889
	GraphRAG	465	30/0	1.86 × 10^-9^	0.918
	LightRAG	435	29/0*	3.73 × 10^-9^	0.947
Consistency	Naive RAG	465	30/0	1.86 × 10^-9^	0.971
	GraphRAG	465	30/0	1.86 × 10^-9^	0.963
	LightRAG	465	30/0	1.86 × 10^-9^	0.942
Clinical utility	Naive RAG	465	30/0	1.86 × 10^-9^	0.939
	GraphRAG	465	30/0	1.86 × 10^-9^	0.959
	LightRAG	435	29/0*	3.73 × 10^-9^	0.922

* 29 wins, 0 losses, 1 tie. The tie is excluded per standard Wilcoxon practice, yielding n' = 29. All 30 cases were evaluated

(3) Cross-paradigm consistency

Comparing the two evaluation paradigms, MAGR-TCM consistently ranked first in both RAGAS and expert evaluation, although the relative rankings of the baseline models differed. The RAGAS metrics yield a wider range of effect sizes (r = 0.559–0.998) than the expert metrics (r = 0.889–0.971), reflecting the greater granularity of automated continuous scoring compared to the bounded 0–1 human rating scale. Despite this methodological difference, all comparisons between MAGR-TCM and the baseline models remain statistically significant across both evaluation frameworks, providing robust evidence for MAGR-TCM's superiority.

#### Safety stress-test results

To independently evaluate the safety performance of the system, we conducted a double-blind assessment on Cohort B (30 cases, including 10 Red Flag emergency scenarios), covering three safety-critical metrics: Sensitivity, Specificity, and False Negative Rate (FNR). The results are presented in Table 10.

**Table 10:** Safety stress-test results on Cohort B

Model	Sensitivity	Specificity	FNR
MAGR-TCM	1.000	1.000	0.000
LightRAG	N/A	1.000*	1.000
Naive RAG	N/A	1.000*	1.000
GraphRAG	N/A	1.000*	1.000

*The Specificity of 1.000 for the baseline models is a mathematical artifact of never triggering an alarm, as they lack any emergency triage mechanism

Baseline models lack dedicated emergency detection mechanisms. Their Specificity of 1.000 merely reflects the vacuum state of never triggering an alarm, and should not be misinterpreted as safe clinical performance. A system that never raises an alarm has no false positives by definition—but also no true positives. Their Sensitivity is marked as N/A (Not Applicable) rather than 0.000, as these baseline models lack any dedicated emergency detection module. This reflects an architectural design absence rather than a technical failure: all emergency cases were routed to routine TCM consultation without triggering any safety alarm.

Across the 30-case cohort (10 emergency + 20 routine cases), MAGR-TCM’s Red Flag mechanism correctly intercepted all 10 emergency cases and properly passed through all 20 routine cases, achieving Sensitivity = 1.000, Specificity = 1.000, and FNR = 0.000. In contrast, the baseline models (Naive RAG, LightRAG, GraphRAG) lack any emergency triage mechanism. Consequently, they either erroneously force a routine TCM diagnosis (e.g., attempting to treat severe acute abdominal pain with standard herbal formulas) or rely on the unpredictable safety refusals of the underlying LLM—an approach that is insufficiently reliable for a medical triage system. This is not merely a difference in technical performance, but a fundamental difference in safety design: one model has a dedicated emergency kill switch, while the others do not.

#### Latency analysis

For home health consultation scenarios, system response time is a practical deployment consideration. We measured the end-to-end latency of all four models on the combined 60 clinical case records from Cohort A and Cohort B under identical hardware and network cond

**Table 11:** End-to-end latency across models and agent-level breakdown for MAGR-TCM

Model	Sub-module / Agent	Mean (s)	SD (s)
Naive RAG	—	13	4.2
LightRAG	—	19	15.7
GraphRAG	—	28.8	12.3
MAGR-TCM	Total	28.0	4.1
	Agent 1	5.3	0.6
	Agent 2	1.7	0.2
	Agent 3	8.5	2.1
	Agent 4	12.5	3.2

itions. The results are summarized in Table 11.

Naive RAG exhibited the lowest latency (13.0 ± 4.2 s), as it requires only a single retrieval-generation call. LightRAG (19.0 ± 15.7 s) introduces a keyword extraction step, increasing latency with high instability—the standard deviation reaches 83% of the mean, with the slowest individual cases exceeding 50 s. GraphRAG (28.8 ± 12.3 s) incurs further overhead from three-stage serial calls (entity extraction, community summary, generation).

MAGR-TCM recorded a mean latency of 28.0 ± 4.1 s. Agent-level decomposition, in execution order, reveals the following internal structure. Agent 1 (Question Analysis, 5.3 ± 0.6 s, 19%) performs four sequential sub-tasks—emergency triage pre-scan, medical terminology correction, 11-category entity extraction, and KG semantic mapping validation—each requiring a dedicated LLM call. Its low SD (0.6 s) reflects the fixed-length nature of these pre-processing prompts. Agent 2 (Routing + Triage, 1.7 ± 0.2 s, 6%) executes a single routing decision and deterministic entity anchoring, resulting in the lowest absolute latency and minimal variability among the four agents. Agent 3 (Graph Reasoning, 8.5 ± 2.1 s, 30%) carries the core computational burden of the 4-hop BFS traversal over the KG and the subsequent LLM-based evidence synthesis into a structured diagnostic report. Agent 4 (Evaluation + Optimization, 12.5 ± 3.2 s, 45%) is the dominant contributor to both mean latency and variability. It performs quality evaluation and, in approximately 10% of cases, triggers full dual-model optimization, creating a bimodal latency distribution that accounts for its larger SD. The root-sum-square of the four agent SDs closely matches the total SD of 4.1 s, indicating that agent-level latencies are approximately independent, with each agent contributing its own variability to the pipeline.

MAGR-TCM's higher absolute latency relative to Naive RAG reflects the computational investment of its multi-agent pipeline: Agent 3's KG reasoning and Agent 4's quality evaluation directly underpin the system's high Faithfulness and Clinical Utility. At the same time, MAGR-TCM achieved the lowest Coefficient of Variation (CV = 0.15) among all models, substantially outperforming LightRAG (CV = 0.83). This response-time consistency is critical for home health deployment: users can expect a predictable waiting time of approximately 28 seconds regardless of case complexity, whereas LightRAG's highly variable latency—ranging from under 10 seconds to over 50 seconds—undermines user trust and practical reliability. MAGR-TCM thus trades a moderate and consistent computational overhead for rigorous safety verification and robust hallucination suppression, making it well-suited for asynchronous home health consultation.

#### Hop-depth ablation: structural completeness vs. retrieval efficiency

To empirically determine the optimal reasoning depth sufficient for complete TCM prescription generation, we conducted a hop-depth ablation on the 30-case Cohort A test set, measuring entity-type coverage and cumulative path cost across 2 to 5 hop configurations. Table 12 reports the coverage achieved at each depth alongside the marginal path cost per additional percentage point of coverage.

**Table 12:** Hop-depth ablation: entity-type coverage and marginal path cost

Hop	Coverage	Paths	Cases at 100%	Δ Coverage	Δ Paths	Cost/1% Cov
2-hop	0.707	89	0/30	—	—	—
3-hop	0.933	417	23/30	+0.226	+328	15
4-hop	1.000	1085	30/30	+0.067	+668	100
5-hop	1.000	4579	30/30	+0.000	+3494	N/A

A dedicated hop-depth validation experiment on the 30-case Cohort A test set empirically validated this design choice. Across hop depths 2 to 5, entity type coverage progressed from 0.707 at 2 hops, where no case achieved full coverage (0/30), to 0.933 at 3 hops, where 7 cases remained incomplete (23/30 full coverage), to 1.000 at 4 hops, where all 30 cases achieved complete coverage (30/30). While transitioning from 3 to 4 hops requires a marginal path cost of 100 paths per 1% coverage gain, the coverage reaches its absolute structural ceiling at 4 hops. At 5 hops, the marginal coverage increment drops to zero—the additional 3,494 paths generated at this depth yield no further structural benefit, confirming that 4 hops represents the definitive efficiency optimum. Thus, 4 hops is the minimum depth required for complete TCM prescription generation, beyond which additional graph traversals are clinically and computationally unjustifiable.

## Discussions

### Analysis of model effectiveness

The experimental results demonstrate that MAGR-TCM outperforms existing baseline models across both automated and human evaluation metrics. This superior performance can be attributed to the model's unique architecture. First, regarding Faithfulness, MAGR-TCM's high score is driven by its path constraint mechanism, which mitigates hallucinations by anchoring generation to retrieved KG evidence.. In contrast, while GraphRAG captures global information, it suffered fidelity loss due to challenges in aligning abstract summaries with specific retrieved segments. Naive RAG's lowest faithfulness score confirms that its fragmented retrieval mechanism tends to erroneously splice information, lacking the structured constraints necessary for medical safety. Second, in terms of Answer Correctness and Pattern Differentiation, MAGR-TCM leverages explicit path reasoning along the Knowledge Graph to anchor the "Symptom → Pathogenesis" linkage. This allows it to capture implicit clues that statistical models miss. Notably, while GraphRAG achieved relatively high Answer Similarity by generating stylistically relevant medical text, its Answer Correctness scored poorly (0.495). This discrepancy occurs because the RAGAS Answer Correctness metric strictly penalizes factual inconsistencies—such as recommending an incorrect formula—which MAGR-TCM successfully avoids through its graph-based constraints. Furthermore, although Naive RAG showed high Answer Similarity, its low Faithfulness suggests that this similarity stems primarily from surface keyword overlap rather than correct underlying logic. LightRAG, while faster, lacks dedicated combinatorial rules, often conflating correlation with causation. Third, regarding Clinical Utility, the Multi-Agent collaboration ensures that MAGR-TCM generates not only formulas but also verification evidence ("Differentiation Basis"). This transparency is crucial for clinical adoption, whereas GraphRAG’s broad summaries often lacked the granular detail required for specific prescription guidance.

MAGR-TCM exhibited the lowest per-case variance among all models (σ^2^ = 0.0011–0.0013), with individual case scores tightly clustered within a narrow band: across all 30 cases, Pattern Differentiation Accuracy never fell below 0.80, Consistency never fell below 0.77, and Clinical Utility never fell below 0.83. This confirms that MAGR-TCM's performance is not only superior on average but also remarkably consistent—it does not produce occasional low-quality outputs on difficult cases, a critical requirement for unsupervised home health deployment. In contrast, Naive RAG showed the highest variance (σ^2^ = 0.0253–0.0803), with individual case scores spanning from 0.00 to 0.83. Scores of 0.00 occurred when Naive RAG's retrieval module found no relevant document chunks and the model responded with "I don't know"—a complete failure mode absent from the graph-based models. The wide score distribution reflects Naive RAG's fundamental dependence on whether the retrieval module happened to return relevant content for a given case: on simple cases with straightforward symptom descriptions, it occasionally achieved scores as high as 0.83 (Pattern Differentiation Accuracy), but these instances were sporadic and unpredictable. GraphRAG exhibited intermediate variance (σ^2^ = 0.0167–0.0328), with scores ranging from 0.20 to 0.80. Its community-summary mechanism provides more stable performance than Naive RAG's unstructured retrieval, but the macro-level summaries can miss case-specific nuances (e.g., conflating two syndromes that share similar terminology but differ in cold/heat attribution), leading to moderate score fluctuations. LightRAG's variance approached MAGR-TCM's (σ^2^ = 0.0023–0.0035), indicating similarly stable output quality. However, its mean scores (0.700–0.734) were substantially lower than MAGR-TCM's (0.884–0.898), with a gap of approximately 0.15–0.18 across all three dimensions. This pattern—stable but consistently lower—reveals a fundamental architectural limitation: LightRAG's simplified entity co-occurrence retrieval reliably produces acceptable outputs but lacks the structured multi-hop reasoning depth to reach the diagnostic precision and completeness of MAGR-TCM. Put differently, LightRAG is consistently adequate; MAGR-TCM is consistently excellent.

### Limitations

Despite the promising results, this study has four notable limitations that should be acknowledged. (1) Dataset Constraints: Constrained by the difficulty of acquiring high-quality TCM clinical cases, the current dataset size of 741 cases is relatively limited. This may restrict the model's generalization capabilities when encountering rare patterns or atypical symptom combinations not covered in the training set. (2) Lack of Multimodal Integration: The current KG construction is primarily based on textual representations and has not yet deeply integrated objective quantitative indicators from modern medicine (e.g., ultrasound imaging or serological markers). This absence limits the model's comprehensive decision-making precision in integrated TCM-Western medicine diagnostic scenarios. (3) Diversity of TCM Schools of Thought: TCM is characterized by diverse academic lineages (e.g., the Jingfang School and the Wenbing School), wherein practitioners may exhibit varying diagnostic perspectives and prescribing habits for identical clinical presentations. As our current model is trained on consensus logic and standard textbooks, it may not fully capture the personalized nuances and flexible modifications specific to disparate TCM schools of thought. (4) Lack of Prospective Real-World Validation: crucially, all evaluations in this study were retrospective, conducted on expert-annotated clinical case records under controlled conditions. Consequently, the reported performance metrics should be interpreted as upper-bound estimates. The system has not yet undergone prospective validation with real-world home users, whose spontaneous inputs may contain typos, vague colloquial descriptions, and mixed-language expressions that differ substantially from curated case narratives. Furthermore, while the Red Flag mechanism achieved outstanding performance on Cohort B, formal validation against clinical emergency triage standards (e.g., Emergency Severity Index) remains warranted. Although this retrospective approach is ethically appropriate and scientifically sufficient for the current scope, large-scale prospective cohort studies in real home settings are essential to further validate its long-term clinical safety, operational resilience under colloquial language, and health economic value.

## Conclusions

Addressing the critical challenges of knowledge implicitness inherent in TCM gynecology and generative hallucinations typical of large language models, this paper proposes MAGR-TCM, an intelligent home health consultation and triage model that integrates a domain-specific Knowledge Graph with a Multi-Agent collaborative mechanism. The core contribution of this study lies in dismantling the traditional "Black Box Retrieval" paradigm, transforming unstructured, tacit clinical experience into transparent and interpretable logical reasoning paths. Through a rigorous closed-loop workflow, the model demonstrates superior performance over baseline methods in faithfulness, clinical utility, response stability, and emergency triage accuracy.

To unlock greater clinical and societal value, future research will aim for breakthroughs in: (1) vertically expanding the disease spectrum to encompass refractory gynecological conditions; (2) leveraging natural language processing to mine ancient TCM texts—particularly focusing on regional lineages like Xin'an Medicine—to enrich the knowledge base; (3) incorporating Western medical knowledge to construct an integrated TCM-Western Medicine Knowledge Graph; and (4) conducting large-scale prospective cohort studies in real home settings to validate the system's long-term clinical safety, operational resilience under colloquial language input, and health economic value.

## Data Availability

The datasets analyzed during the current study are derived from the 'Ancient and Modern Medical Case Cloud Platform' (https://www.yiankb.com/home), maintained by the Institute of Information on Traditional Chinese Medicine, China Academy of Chinese Medical Sciences.

## Abbreviations

MAGR‑TCM: Multi‑Agent Graph Retrieval‑Augmented Generation for Traditional Chinese Medicine
TCM: Traditional Chinese Medicine
LLM: Large Language Model
RAG: Retrieval‑Augmented Generation
GraphRAG: Graph‑enhanced Retrieval‑Augmented Generation
LightRAG: Lightweight Retrieval‑Augmented Generation
GPT-4: Generative Pre-trained Transformer 4
KG: Knowledge Graph
PCOS: Polycystic Ovary Syndrome
GBD: Global Burden of Disease Study
NER: Named Entity Recognition
RAGAS: Retrieval‑Augmented Generation Assessment
CI: Confidence Interval
CV: Coefficient of Variation
FNR: False Negative Rate
ESI: Emergency Severity Index
